# Comparison of periodontal parameters between patients with ischemic and dilative cardiomyopathy

**DOI:** 10.1186/s12872-021-02111-5

**Published:** 2021-06-16

**Authors:** Dirk Ziebolz, Christian Binner, Florentine Reuschel, Mirjam Eisner, Justus Wagner, Tanja Kottmann, Christian D. Etz, Sven Lehmann, Jens Garbade, Gerhard Schmalz

**Affiliations:** 1 Department of Cariology, Endodontology and Periodontology, University of Leipzig, Liebigstr. 12, 04103 Leipzig, Germany; 2 University Department of Cardiac Surgery, Heart Center Leipzig, Leipzig, Germany; 3 CRO Dr. med. Kottmann GmbH & Co. KG, Hamm, Germany; 4 Department of Cardiac Surgery, Klinikum Links der Weser, Bremen, Germany

**Keywords:** Heart failure, Periodontitis, Periodontal medicine, Ischemic heart disease

## Abstract

**Background:**

This cross-sectional study aimed in the comparison of periodontal parameters, number of remaining teeth and oral behaviour between patients with ischemic- (ICM) and non-ischemic dilative cardiomyopathy (DCM).

**Methods:**

Patients with HF from the Department for Cardiac Surgery at the Heart Center Leipzig were included. The two groups (ICM and DCM) were composed by matching according to age, gender and smoking habits. All participants received a comprehensive periodontal examination, including a periodontal probing on six measurement points of each tooth.

**Results:**

A total of 226 patients (n = 113 each group) was included. Patients in DCM group used interdental cleaning significantly more often than ICM (23.9% vs. 12.5%, *p* = 0.04). The majority of patients in both groups (ICM: 83.6%, DCM: 84.6%, *p* = 0.23) were diagnosed with stage III–IV periodontitis. Periodontal parameters were comparable between groups (*p* > 0.05). Variance analysis revealed no influence of the group (ICM vs. DCM) on the number of remaining teeth (*p* = 0.16), periodontitis stage (*p* = 0.27) or the periodontal inflamed surface area (*p* = 0.62).

**Conclusions:**

Patients with severe HF show high periodontal burden, without any differences between ICM and DCM group. Therefore, increased attention should be payed to periodontal health of patients with severe heart disease, irrespective of their underlying disease.

## Introduction

Heart Failure (HF) is an important cause of morbidity and mortality and therefore a relevant area of recent research activities [1]. During disease progress of HF, circulatory support, e.g. in form of left ventricular assist device (LVAD) becomes frequently necessary; thereby, heart transplantation (HTx) remains an important therapy option in individuals suffering from end-stage HF [2]. Underlying diseases for HF can be various, including cardiac conditions, hereditary defects and systemic diseases [3]. One important cause of HF are ischemic heart diseases, especially ischemic cardiomyopathy (ICM) [3, 4]. This aetiology is primarily related to coronary artery diseases, which are characterized by formation of plaques in coronary vessels, leading to reduced blood circulation and thus supply of the contractile heart muscle [4].

Periodontitis, as an inflammatory disease of the periodontal tissues has been repeatedly reported to be associated to cardiovascular diseases, especially in context of coronary heart diseases [5, 6]. Thereby, different potential biological pathways have been identified, which could explain an influence of periodontal inflammation on atherogenesis [7]. Against this background, the role of periodontal therapy on the prevention of the formation and/or progression of cardiovascular diseases has been discussed; however, there is only very low quality of evidence on this clinical issue [8]. Thus, the causality and clinical relevance of the interrelationship between periodontal and cardiovascular diseases, including coronary heart diseases are still questionable.

Patients with advanced HF, especially if treated with either LVAD or HTx were recently reported to show a lack in periodontal care [9–11]. Thereby, a high periodontal treatment need, alongside with more severe periodontal disease burden was found in these individuals [9–11]. This might be hazardous, because this patient group is a vulnerable clientele, especially if treated with an HTx [12]. However, it has up until now not been examined, whether this phenomenon is primarily observed in individuals with ischemic heart diseases or if this is a general problem in patients with HF. Based on the potential relationship between periodontal diseases and coronary heart diseases, a higher prevalence and severity of periodontitis might be conceivable in patients with ICM.

Therefore, this clinical cross-sectional study aimed in the comparison of periodontal parameters, number of remaining teeth and oral behaviour between patients with ICM and with non-ischemic dilative cardiomyopathy (DCM). As periodontal parameters, clinical periodontal findings, diagnosis (stage and grade of periodontitis) and the quantitative periodontal inflammatory surface were assessed. It was hypothesized that patients with underlying ICM would show worse periodontal conditions compared to DCM patients.

## Methods

This study was based on a monocentric cross-sectional study in cooperation of the Department for Cardiac Surgery, Heart Center Leipzig and the Department of Cariology, Endodontology and Periodontology, University of Leipzig. All examinations were performed in full accordance with the Declaration of Helsinki and the study protocol was reviewed and approved by the local ethics committee of the Medical Faculty of University of Leipzig (No: 414/16-ek). All participants were informed verbally and in writing about the study and gave their informed consent.

### Patients

Between May 2017 and December 2018, patients, who attended the Department for Cardiac Surgery, Heart Center Leipzig for routine follow-up appointment because of either HF, HTx or LVAD underwent a full oral examination. Prior to dental and periodontal examination, patients were informed about the study and provided written informed consent for participation. Out of this patient group, individuals were included in this current study based on previously defined in- and exclusion criteria. Inclusion criteria were age ≥ 18 years, the ability to provide informed consent and the presence of either ICM or DCM as causal underlying disease. The following exclusion criteria were formulated:

- Worse general health status making oral examination impossible
- Autoimmune diseases (e.g. rheumatoid arthritis)
- Infectious diseases (hepatitis A, B, C, tuberculosis, HIV)
- Pregnancy

Two groups were composed, based on the fact whether ICM or DCM was a causal underlying disease of the patients. To ensure comparability, the groups were composed by a matching according to age, gender and smoking habits. Based on the medical records, several general health and cardiological data, smoking habits (smoker: currently smoking, former smoker: smoking within five years before examination, non-smoker: no smoking for at least five years), age and gender, co-morbidities and different disease/therapy-related parameters were collected.

### Questionnaires

Standardized questionnaires were applied to each patient to assess their oral hygiene (tooth brush, interdental cleaning) and dental behavior (regular dental visits, regular professional tooth cleaning). Moreover, a pattern of periodontal complaints, especially the presence of bleeding, tooth mobility, halitosis or bad taste was asked.

### Oral examination

All oral examinations were performed at the Department for Cardiac Surgery, Heart Center Leipzig, by three experienced and calibrated (kappa > 0.8) dentists under standardized conditions. Participants after HTx and with LVAD received 2 g Amoxicillin (or 600 mg Clindamycine in case of an allergy against penicillin) as an antibiotic prophylaxis an hour prior to examination [13]. All patients underwent a general oral examination in a visual manner using mirror and probe to record the number of remaining teeth, remaining molars/premolars and front teeth, respectively. A complete periodontal examination was performed for each dentate patient, whereby periodontal probing depth (PPD), clinical attachment loss (CAL) and bleeding on probing (BOP) were recorded at six measurement points per tooth with a millimeter scaled periodontal probe (PCP 15, Hu-Friedy, Chicago, IL, USA). The findings of periodontal examination lead to a diagnosis based on the current classification of periodontal diseases, within the available staging (stage I–IV) and grading (grade: A–C) matrix as follows[14]:

- Stage I: interdental CAL max. 1-2 mm
- Stage II: interdental CAL max. 3-4 mm
- Stage III: interdental CAL max. ≥ 5 mm, periodontitis-related tooth loss ≤ 4 teeth
- Stage IV: interdental CAL max. ≥ 5 mm, periodontitis-related tooth loss ≥ 5 teeth
- Grade A: bone loss/age < 0.25
- Grade B: bone loss/age 0.25–1.0 and/or smoking < cigarettes/day and/or diabetes mellitus with HbA1c < 7.0%
- Grade C: bone loss/age < 1.0 and/or smoking ≥ 10 cigarrettes/day and/or diabetes mellitus with HbA1c ≥ 7%

Furthermore, the periodontal inflamed surface area (PISA) was determined based on inflamed periodontal pockets to quantify the inflammatory periodontal burden, as described by Nesse et al. [15].

### Statistical analysis

The statistical analysis was performed with SPSS for Windows, version 24.0 (SPSS Inc., US). Prior to analysis, Kolmogorov–Smirnov-test was applied to assess whether parameters were normal distributed. Thereby, non-normal distribution was found for the examined metric variables (*p* < 0.05). Accordingly, Mann–Whitney-U test as non-parametric test was used for metric variables. Additionally, either chi-square or fisher test were used to analyse categorical or nominal data, respectively. For multivariate analysis, a variance analysis was applied, considering number of remaining teeth, periodontitis stage and PISA as the dependent variable. Therefore, the group (ICM or DCM) was considered as main effect and the influential factors age, gender and smoking habits were chosen. A two-sided significance testing was used for all analyses, whereby the significance level has been set at *p* < 0.05.

## Results

### Patients

In each group (ICM and DCM), a total of 113 patients with comparable age, gender and smoking habits were included (*p* > 0.05). The majority of participants were treated by LVAD, without differences between ICM and DCM (*p* = 0.50). Moreover, ejection fraction, co-morbidities, medication as well as laboratory values were comparable between ICM and DCM group (*p* > 0.05, Table 1).

**Table 1:** Patient characteristics

	ICM (n = 113)	DCM (n = 113)	*p* value
Gender (female in % [n])	7.1% [8]	7.1% [8]	0.99
Age in years (mv ± sd)	59.55 ± 7.61 [60.0, 55.0–65.0]	59.54 ± 9.00 [61.0, 54.0–66.0]	0.86
Smoking habits % [n]			
Smoker	15.9% [18]	15.9% [18]	0.99
Non-smoker	61.1% [69]	61.1% [69]	
Former smoker	23% [26]	23% [26]	
Co-morbidities % [n]			
Hypertension	73.5% [83]	69.9% [79]	0.66
Diabetes mellitus	47.8% [54]	41.6% [47]	0.42
Renal insufficiency	61.9% [70]	62.8% [71]	0.99
Osteoporosis	1.8% [2]	3.5% [4]	0.68
Ejection fraction	34.61 ± 16.80 [30.0, 23.0–53.0]	32.84 ± 16.47 [26.9, 20.0–48.0]	0.17
Disease/Therapy situation			
HTx	29.2% [33]	23.9% [27]	0.50
LVAD	46.0% [52]	45.1% [51]	
HF	24.8% [28]	31% [35]	
Medication			
ACE-blocker	84.7% [94/111]	78.6% [88/112]	0.30
Calcium channel clocker	18.6% [21/111]	14.3% [16/112]	0.37
Anticoagulants	64% [71/111]	68.8% [77/112]	0.48
Antiplatelet medication	82.9% [92/111]	75.9% [85/112]	0.25
Immunosuppressive	31.5% [35/111]	26.8% [30/112]	0.46
Insulin	23.4% [26/111]	17% [19/112]	0.25
Oral antidiabetic drugs	19.8% [22/111]	17.9% [20/112]	0.74
Bisphosphonates	11.7% [13/111]	14.3% [16/112]	0.69
Laboratory values			
CRP (mg/dl)	9.43 ± 18.94 [3.7, 1.7–7.8]	13.40 ± 24.64 [3.8, 1.6–16.0]	0.81
Creatinine (µmol/l)	64.40 ± 112.41 [20.9, 1.2–96.6]	51.81 ± 120.31 [20.4, 1.1–77.0]	0.21
Haemoglobin (mmol/l)	8.44 ± 1.69 [8.6, 7.8–9.1]	7.84 ± 1.97 [8.0, 6.8–9.1]	0.13
Erythrocytes (10^6^/µl)	4.51 ± 0.66 [4.5, 4.1–5.0]	4.41 ± 0.82 [4.5, 3.8–5.0]	0.61
Leukocytes (10^3^/µl)	7.57 ± 2.03 [7.9, 6.3–8.3]	7.12 ± 2.73 [6.6, 5.2–8.3]	0.13
Platelet count (10^3^/µl)	219.19 ± 79.20 [199.5, 169.0–268.0]	231.82 ± 66.92 [225.5, 186.0–280.0]	0.21

Values are given either as mv ± sd [median, IQR] or as % [n]

ICM, ischemic cardiomyopathy; DCM, dilatative cardiomyopathy; HTX, heart transplantation; LVAD, left ventricular assist device; HF, heart failure; CRP, c-reactive protein; mv, mean value; sd, standard deviation; IQR, inter quartile range

### Questionnaires

Results of oral hygiene behaviour and periodontal complaint questionnaires are given in Table 2. About two third of participants reported to undergo regular dental treatment (*p* = 0.77). While in both groups the majority of participants used a manual toothbrush for oral hygiene (ICM: 71.4%, DCM: 73.5%, *p* = 0.77), patients in DCM group used interdental cleaning significantly more often (23.9% vs. 12.5%, *p* = 0.04). Periodontal complaints were comparable between groups (*p* > 0.05, Table 2).

**Table 2:** Findings of oral hygiene and periodontitis questionnaire

Symptoms	ICM	DCM	*p* value
Regular dental treatment % [n]	69.7% [76/109]	67.3% [76/113]	0.77
Regular professional tooth cleaning % [n]	36.6% [41/112]	34.5% [39/113]	0.78
Full dental clearance/rehabilitation % [n]	55% [60/109]	53.2% [59/111]	0.79
Tooth brush % [n]			
Manual	71.4% [80/112]	73.5% [83/113]	0.77
Power	28.6% [32/112]	26.5% [30/113]	
Interdental cleaning % [n]	12.5% [14/112]	23.9% [27/113]	**0.04**
Bleeding gums % [n]	15.9% [18/113]	13.3% [15/113]	0.71
Tooth mobility % [n]	15.3% [17/111]	7.2% [8/111]	0.88
Tooth migration % [n]	18.8% [21/112]	11.6% [13/112]	0.19
Swollen gums % [n]	9.8% [11/112]	5.4% [6/112]	0.31
Halitosis % [n]	14.7% [16/109]	17.1% [19/111]	0.71
Bad taste % [n]	18% [20/111]	25% [28/112]	0.25
Aching gums % [n]	9.8% [11/112]	9.7% [11/113]	0.99
Previous periodontitis therapy % [n]	11.1% [12/108]	18.2% [20/110]	0.21
Recession % [n]	25% [28/112]	24.5% [27/110]	0.99
Periodontal complaint-oriented investigation % [n]	30.9% [34/110]	23.6% [26/110]	0.29

ICM, ischemic cardiomyopathy; DCM, dilatative cardiomyopathy

Significant values (*p* < 0.05) are highlighted in bold

### Oral examination

#### Remaining teeth

A total of 8% of patients in each group were edentulous. Overall, participants were found to have on average 19.00 ± 8.90 [22.0, 14.0–25.0] (ICM) and 17.29 ± 9.11 [20.0, 10.0–25.0] (DCM) teeth, respectively (*p* = 0.17). The number of remaining molars/premolars as well as front teeth was not significantly different between ICM and DCM (*p* > 0.05, Table 3).

**Table 3:** Oral and periodontal findings

Parameter	ICM	DCM	*p* value
Edentolous	8% [9/113]	8% [9/113]	0.99
Remaining teeth (mv ± sd)	19.00 ± 8.90 [22.0, 14.0–25.0]	17.29 ± 9.11 [20.0, 10.0–25.0]	0.17
Remaining molars/premolars (mv ± sd)	9.62 ± 5.26 [11.0, 5.0–14.0]	8.50 ± 15.54 [9.0, 3.0–14.0]	0.15
Remaining front teeth (mv ± sd)	9.42 ± 4.08 [12.0, 8.0–12.0]	8.70 ± 4.06 [11.0, 6.0–12.0]	0.06
Number of teeth CAL ≥ 5 mm (mv ± sd)	4.84 ± 5.26 [3.0, 0–8.0]	5.17 ± 5.37 [4.0, 1.0–8.0]	0.49
Number of teeth PPD ≥ 5 mm (mv ± sd)	6.43 ± 6.42 [4.0, 1.0–10.0]	5.77 ± 5.71 [4.0, 1.0–9.0]	0.49
PPD mean in mm (mv ± sd)	2.96 ± 0.66 [2.9, 2.5–3.4]	2.98 ± 0.66 [2.8, 2.6–3.3]	0.90
CAL mean in mm (mv ± sd)	2.77 ± 1.05 [2.4, 2.0–3.4]	3.15 ± 1.35 [2.8, 2.1–3.7]	0.06
BOP %	18.38 ± 15.81 [17.0, 7.0–26.0]	22.16 ± 21.89 [14.0, 7.0–30.5]	0.78
Periodont-itis grade % [n]			
B	82.7% [86/104]	91.3% [95/104]	0.10
C	17.3% [18/104]	8.7% [9/104]	
PISA in mm^2^ (mv ± sd)	267.85 ± 306.05 [180.5, 74.8–402.9]	253.30 ± 276.43 [161.8, 57.7, 307.6]	0.53
Gingival overgrowth % [n]	7.1% [8/113]	2.7% [3/113]	0.22
PBI in % (mv ± sd)	11.68 ± 14.31 [8.0, 0–19.0]	12.35 ± 13.95 [8.0, 0–20.0]	0.74
Periodontitis stage % [n]			
I	1% [1]	0% [0]	0.23
II	15.4% [16]	15.4% [16]	
III	36.5% [38]	27.9% [29]	
IV	47.1% [49]	56.7% [59]	

Values are given either as mv ± sd [median, IQR] or as % [n]

ICM, ischemic cardiomyopathy; DCM, dilatative cardiomyopathy; CAL, clinical attachment loss; PPD, periodontal probing depth; BOP, bleeding on probing; PBI, papilla bleeding index; mv, mean value; sd, standard deviation; IQR, inter quartile range

#### Periodontal findings

The assessed periodontal parameters were comparable between ICM and DCM group (*p* > 0.05, Table 3). In general, the majority of patients in both groups (ICM: 83.6%, DCM: 84.6%, *p* = 0.23) were diagnosed with stage III-IV periodontitis. The PISA was determined to be on average 267.85 ± 306.05 [180.5, 74.8–402.9] (ICM) and 253.30 ± 276.43 [161.8, 57.7–207.6] (DCM), respectively (*p* = 0.53, Table 3).

#### Influence of remaining teeth, periodontitis stage and PISA on disease group

Variance analysis revealed no influence of the group (ICM vs. DCM) on the number of remaining teeth (*p* = 0.16), periodontitis stage (*p* = 0.27) or the PISA (*p* = 0.62, Table 4). Only gender was found to influence the determined PISA (β: − 168, CI95: − 9.7 to − 326.3; *p* = 0.04), showing female participants to have a higher PISA than males.

**Table 4:** Variance analysis of oral and periodontal conditions

	Remaining teeth				Periodontitis stage				PISA			
	β	CI95 upper	CI95 lower	*p* value	β	CI95 upper	CI95 lower	*p* value	β	CI95 upper	CI95 lower	*p* value
Group ICM/DCM	1.7	4.1	− 0.7	0.16	− 0.1	0.1	− 0.3	0.27	19.8	99	− 59.3	0.62
Age	− 0.1	0.1	− 0.2	0.52	0.1	0.1	0	0.06	0.2	5.1	− 4.6	0.92
Gender	1.7	6.3	− 3.0	0.48	− 0.1	0.4	− 0.5	0.85	− 168	− 9.7	− 326.3	**0.04**
Smoking	0.1	3.4	− 3.3	0.98	− 0.1	0.2	− 0.4	0.50	− 73.4	37.9	− 184.8	0.20

PISA, periodontal inflamed surface area; ICM, ischemic cardiomyopathy; DCM, dilatative cardiomyopathy

Significant values are highlighted in bold (*p* < 0.05)

## Discussion

### Summary of the main results

The majority of patients had a severe periodontitis (stage III-IV), irrespective of the underlying disease group (ICM or DCM). There was no group effect of ICM/DCM on the number of remaining teeth, periodontitis stage or PISA.

### Comparison with published data

This is the first study, which compared periodontal findings between patients with ICM and DCM. Generally, periodontitis or periodontal treatment need is an important public health issue, regardless of the presence of a severe heart disease; the population-representative fifth German oral health study (DMS V) showed about three quarters of German older adults to have periodontal treatment need [16]. Especially in the old Germans, periodontal diseases are reported to be underscreened, underdiagnosed and thus often undertreated [17]. Alongside with the potential relationship between periodontal and cardiovascular diseases [5], a high periodontal burden in severely heart diseased patients appears plausible. The high periodontal burden and treatment need of patients with severe HF, after LVAD and/or HTx has been reported previously and appears an expectable finding [9–11].

This current study focused on the comparison between ICM and DCM group regarding periodontal parameters and thereby it was hypothesized that patients with underlying ICM would show worse periodontal conditions compared to DCM patients. This hypothesis originated from the deliberation that ischemic heart diseases are most likely related to atherosclerotic processes of the heart vessels, which have been repeatedly reported to be associated with periodontal diseases [5, 7]. Although this seems plausible, the current study did not confirm any group effect; however, the prevalence of severe periodontitis (stage III and IV) was very high in both groups. This might be explained by different approaches. On the one hand, the causality between cardiovascular and periodontal diseases is still questionable [18]. Even if there is an independent effect of periodontal diseases on cardiovascular outcomes, as stated in the consensus report [5], there is still limited evidence, especially for the preventive effect of periodontal therapy on cardiovascular disease progress [19]. Thereby it remains conceivable, that the association between periodontal and cardiovascular diseases is primarily caused by shared risk factors (e.g. smoking) and underlying metainflammation and is therefore more coincidental than causal [18]. This might not be different for ischemic and non-ischemic aetiology of the HF and thus could explain that there is no group effect in the current study. A further explanation is the reduced oral hygiene behaviour of the patients with severe HF, irrespective of the group (ICM vs. DCM). Especially the low usage of interdental cleaning devices was thereby conspicuous in the current study. A previous study found oral hygiene to be reduced in patients with cardiovascular diseases, too [20]. Therefore, increased attention should be paid on oral hygiene of patients with heart diseases; however, there is limited evidence for strategies to promote oral hygiene in heart diseased individuals and it remains unclear if there would be an effect on cardiovascular outcome [21]. Another explanation could be the time of investigation, because all patients were in the end-stage of their heart disease (severe HF, LVAD or HTx). Potentially, a causal relationship between periodontal and cardiovascular disease might get manifest in the early stages of cardiovascular diseases. Accordingly, a potential effect of the periodontal disease might be irrelevant in the end-stage of heart disease, because all of the patients are meanwhile severely diseased, irrespective of their underlying pathology. Thereby, in the current study, different parameters of periodontal health were considered, including CAL and periodontitis stage to illustrate the periodontal burden, and the PPD, BOP and PISA for current inflammation, respectively. Especially the PISA was assumed to be a promising parameter to detect a group effect, because it allows quantification of periodontal inflammation [15, 22]. Thereby, PISA is the sum of inflamed periodontal pockets (pockets with positive bleeding on probing) and shows the current inflammatory burden of the patient [15, 22]. While periodontitis severity (stage and grade) or CAL only reflect the periodontal burden in history, i.e. the past destruction of periodontal hard and soft tissues, PISA might therefore show the current activity of periodontitis. Thus, this parameter was assumed to be of particular clinical significance. One available study showed PISA to be related to hypertension in a large-scaled study [23]. The comparable PISA in the studied groups (ICM vs. DCM) argues for no clinically relevant effect of the underlying disease on inflammatory periodontal conditions. While no other studies regarding patients with heart diseases are available, PISA has been examined in context of other systemic diseases. Thereby, PISA was found to be associated to the disease severity in patients with rheumatoid arthritis, as well as to clinical response to medication of rheumatic diseased individuals [24, 25]. Moreover, PISA was shown to be related to increase of CRP in patients with end-stage renal diseases [26]. Accordingly, this parameter seems to be of clinical interest and relevance for patients with systemic diseases. The missing association between PISA and the group (ICM vs. DCM) in the current study is therefore a hint of a low effect of the causal underlying disease on recent periodontal inflammation in the end-stage of heart disease. Moreover, tooth loss (or remaining teeth, respectively) was examined to estimate the previous periodontal burden. It has been reported that tooth loss would be correlated to cardiovascular diseases [27]. However, no associations between underlying disease and remaining teeth was confirmed in the current study, supporting the missing effect of the underlying disease on periodontal health in severely heart diseased individuals.

Altogether, several clinical implications can be striven by the current study´s findings: I) although there could be a causal relationship or association between periodontal and cardiovascular diseases, there seems to be no effect of the causal underlying disease on periodontal parameters in the end-stage of heart disease. II) oral hygiene and dental behaviour needs support in patients with severe HF. III) an early and comprehensive periodontal (and dental) care is needed to support oral health in these individuals. These implications are in line with the consensus conference, recommending an interdisciplinary care for patients with cardiovascular diseases [5]. Similarly, a systematic review concluded frequent dental examinations, tooth cleaning and periodontal therapy to be needed for individuals after HTx [28]. A recent study showed periodontitis to predict an increased risk of ischemic heart disease and death in the long-term follow up [29]. Also another study showed that periodontal inflammation is independently associated with the risk of future cardiovascular events [30]. Thus, reducing periodontal inflammation might also reduce the risk of new cardiovascular events and fatal course in individuals after HTx or with LVAD. Therefore, special dental care concepts might be recommendable for severely heart diseased patients, irrespective of their underlying diseases.

### Strengths and limitations

This is the first study, which investigated periodontal conditions between patients with severe HF, either with underlying ICM or DCM. The sample size was comparably large, with more than 100 participants in each group. Moreover, the groups were matched, and did not differ regarding treatment form (HF, LVAD or HTx), co-morbidities or laboratory values. However, certain heterogeneity, especially caused by the inclusion of LVAD, HTx and HF patients remains a limitation of the study cohort. The different therapy options (HTx or LVAD) are potentially influential factors and might blur an effect of the underlying disease on periodontal outcome. The absence of a healthy control group can be seen as a drawback, because an interpretation of the findings with respect to individuals without heart diseases might be interesting. However, the main objective of this study was to compare patients with underlying ICM and DCM, respectively. For this question, a healthy control would not bring an additional benefit. The high prevalence of periodontitis in both groups is a result of clinical relevance, but complicates finding differences between the groups. Due to the variety of periodontal parameters, even a small effect might have been detected, although the majority of patients was periodontally diseased. The design as a cross-sectional study does not allow any conclusions on a potential influence of periodontal burden or inflammation on the cardiological outcome in the long term.

## Conclusion

Patients with severe HF suffer from a high periodontal disease burden, without differences between ICM and DCM. Periodontal health should be fostered in all of these individuals, irrespective of their underlying disease.

## Data Availability

The datasets generated and/or analysed during the current study are not publicly available due but are available from the corresponding author on reasonable request.

## Abbreviations

CAL: Clinical attachment loss
DCM: Dilatative cardiomyopathy
HF: Heart failure
HTx: Heart transplantation
ICM: Ischemic cardiomyopathy
LVAD: Left ventricular assist device
PBI: Papilla bleeding index
PISA: Periodontal inflamed surface area
PPD: Periodontal probing depth
